# Integrated Leaching and Separation of Metals Using Mixtures of Organic Acids and Ionic Liquids

**DOI:** 10.3390/molecules25235570

**Published:** 2020-11-27

**Authors:** Silvia J. R. Vargas, Helena Passos, Nicolas Schaeffer, João A. P. Coutinho

**Affiliations:** CICECO-Aveiro Institute of Materials, Department of Chemistry, University of Aveiro, 3810-193 Aveiro, Portugal; silvia.vargas@ua.pt (S.J.R.V.); hpassos@ua.pt (H.P.); jcoutinho@ua.pt (J.A.P.C.)

**Keywords:** critical metals, process intensification, metal leaching, solvent extraction, solvometallurgy

## Abstract

In this work, the aqueous phase diagram for the mixture of the hydrophilic tributyltetradecyl phosphonium ([P_44414_]Cl) ionic liquid with acetic acid (CH_3_COOH) is determined, and the temperature dependency of the biphasic region established. Molecular dynamic simulations of the [P_44414_]Cl + CH_3_COOH + H_2_O system indicate that the occurrence of a closed “type 0” biphasic regime is due to a “washing-out” phenomenon upon addition of water, resulting in solvophobic segregation of the [P_44414_]Cl. The solubility of various metal oxides in the anhydrous [P_44414_]Cl + CH_3_COOH system was determined, with the system presenting a good selectivity for CoO. Integration of the separation step was demonstrated through the addition of water, yielding a biphasic regime. Finally, the [P_44414_]Cl + CH_3_COOH system was applied to the treatment of real waste, NiMH battery black mass, being shown that it allows an efficient separation of Co(II) from Ni(II), Fe(III) and the lanthanides in a single leaching and separation step.

## 1. Introduction

Solvometallurgy, the extraction of metals from primary and secondary sources using solutions containing less than 50 vol. % of water, can offer complementary advantages to hydrometallurgical processes. These include the potential integration of the leaching and solvent extraction in a single step as well as a greater leaching selectivity compared to acidic aqueous solutions thereby simplifying subsequent purification steps [1]. For example, simple organic solutions were shown to selectively dissolve gold and platinum group metals with high dissolution rates under mild conditions, thereby providing a more benign alternative to the aggressive aqua regia or cyanidation processes [2,3]. A promising subset of solvometallurgy is ionometallurgy—the processing of metals using solvents containing a high concentration of ions such as ionic liquids (ILs) or deep eutectic solvents (DES) [4]. The important concentration of coordinating anions of most common ionic solvent combined with their low water to ion ratio stabilizes certain metal complexes [5,6] and allows for dissolution reactions and selectivities typically not achievable in aqueous media [7,8,9,10,11].

The versatility and facile preparation of binary mixtures of an ionic compound with an organic acid has resulted in their widespread application as alternative solvents for metal leaching and electrodeposition applications [7,12,13,14,15,16,17,18,19], and to a lesser extent solvent extraction [20,21,22]. Such mixtures were successfully applied to the treatment of real matrices including sulphidic and oxide ores such as pyrite, chalcopyrite or goethite [8,9,10,23], zinc flue dust [11], incinerated sewage sludge ash [24], NdFeB magnets [25], NiMH batteries [26] and lithium-ion batteries [27,28]. Notwithstanding the obvious success of these ionic solvents, a persistent issue remains the selective separation of metals with similar properties and redox potentials, often requiring additional separation steps or their recovery as mixed products [10,11,25,26,29]. Furthermore, it was shown that increasing the complexity of the separation system, can result in the destruction of the mixture integrity and the nonstoichiometric partition of the components between the coexisting phases [30,31]. The development of simple binary mixtures capable of integrating leaching and solvent extraction operations in a single solution by presenting a variable hydrophilic/hydrophobic character with no additional extractant, organic phase or salts could extend the applicability of ionometallurgy. Mixtures of two fully hydrophilic constituents could present additional benefits stemming from the extra degree of tunability provided by small quantities of water in ionic solvents [23,32,33].

Bulky quaternary ammonium and phosphonium ILs such as tetrabutylammonium chloride ([N_4444_]Cl) or tetrabutylphosphonium chloride ([P_4444_]Cl) are common constituents of hydrophilic and hydrophobic mixtures with organic acids despite both salts being fully water-miscible [34]. Most surprisingly for ionic compounds, certain of these hydrophilic quaternary salts including [P_4444_]Cl and its surfactant derivative tributyltetradecylphosphonium chloride ([P_44414_]Cl) present a pronounced lower critical solution temperature (LCST) in the presence of water [35,36,37,38]. The temperature-dependent phase demixing combined with the reported miscibility of quaternary halide salts and numerous organic acids [34] opens up the possibility of reversibly varying the system from monophasic to biphasic through the addition of water and/or temperature manipulation. Such versatile systems could facilitate the integration of leaching and solvent extraction unit operations, providing a bridge between hydrophylic and hydrophobic solvents. Although no such systems were applied to metal recovery, the mixture of lidocaine and oleic acid presents an LCST of 298 K driven by the change in ionicity from oleate to oleic acid with temperature increase. The mixture was shown to efficiently extract a range of dyes from aqueous solutions [39].

In this work, the phase diagram of the IL [P_444414_]Cl with acetic acid (CH_3_COOH) is experimentally and computationally characterized and the mixture applied to the integrated leaching and separation of cobalt from other metals present in Nickel Metal Hydride (NiMH) batteries. Cobalt is a critical metal of high economic importance with demand forecasted to grow due to its inclusion in energy storage technologies, making its recovery from secondary sources increasingly topical [40,41]. Integration of the leaching and separation steps was demonstrated through the simple addition of water, yielding a closed biphasic regime.

## 2. Results and Discussion

### 2.1. Characterisation of the [P_44414_]Cl + CH_3_COOH + H_2_O System

In an effort to identify potential mixtures capable of presenting a variable hydrophilic/hydrophobic character in the absence of an additional salting-out agent, four aqueous solutions of chloride-based ILs ([N_4444_]Cl, [P_4444_]Cl, [P_44414_]Cl and [C_14_mim]Cl) and three organic acids were screened for the existence of a biphasic regime. The quaternary ammonium and phosphonium salts were selected due to their reported temperature-dependent aqueous solubility conferred by the screening of the charge center by the alkyl moieties [35,36,37,38]. Furthermore, comparing the behavior of the symmetric [P_4444_]Cl to its surfactant counterpart [P_44414_]Cl in the presence of organic acids allows to determine the contribution of the IL self-organization on the phase separation. Conversely, the comparison of [P_44414_]Cl with [C_14_mim]Cl permits the evaluation of the surfactant head group, with [C_14_mim]Cl displaying an un-screened charge dispersed around the imidazolium ring. Regarding the biodegradable organic acids, acetic acid, lactic acid and the poly-carboxylic citric acid were studied. These acids were selected due to their reported application both in aqueous solutions and as components in eutectic solvents [13,15,42,43]. It is important to emphasise that the tested acids present a range of acidities, with their deprotonation constant (pKa) ranking as: citric acid (3.13) < lactic acid (3.86) < acetic acid (4.75). The possibility for acid deprotonation and subsequent anion-exchange with the IL’s chloride and the formation of a new and potentially hydrophobic IL must be considered.

From an initial screening of the ternary systems, only mixtures of [P_44414_]Cl with acetic acid were found to form biphasic systems, with all others remaining monophasic under the explored conditions. The screening results hint at three prerequisites for the formation of mixtures presenting variable hydrophilicity. Firstly, of the quaternary ammonium and phosphonium salts tested [P_44414_]Cl is the only one capable of self-assembly into distinct phases in aqueous solution [36,38]. Secondly, a comparison with [C_14_mim]Cl, which did not form any biphasic systems with the tested acids, indicates that IL self-assembly alone is not sufficient. Thirdly, acetic acid possesses a distinct apolar end section and a low deprotonation constant. The possible interactions between CH_3_COOH and [P_44414_]Cl yielding to a biphasic system formation are discussed further in the next sections. There are some structural similarities between the investigated [P_44414_]Cl + CH_3_COOH and the previously-reported [N_4444_]Cl + decanoic acid [44,45], with the long alkyl chain switched from the organic acid to the IL component. However, contrary to the poorly water-soluble decanoic acid, both [P_44414_]Cl and acetic acid are water-miscible.

**Figure 1:**
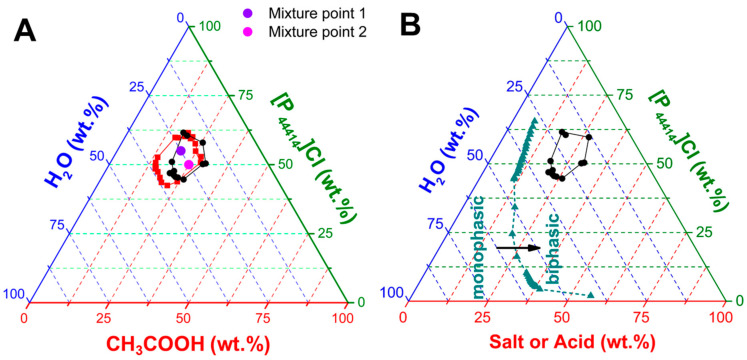
(**A**) Phase diagram of the [P_44414_]Cl + CH_3_COOH + H_2_O system at 298 K (⬤ black) and 323 K (■ red). (**B**) Comparison of the experimental phase diagrams for the aqueous biphasic system composed of [P_44414_]Cl and CH_3_COOH or NH_4_CH_3_COO (▲ cyan) at 298 K.

The aqueous phase diagram of [P_44414_]Cl with CH_3_COOH is presented in Figure 1A. Prior to any discussion, it is important to stress that [P_44414_]Cl is soluble in CH_3_COOH in the absence of water along the entire composition range, yielding a homogenous solution liquid at room temperature. This is markedly different to the reported acid aqueous biphasic systems of [P_44414_]Cl with inorganic acids such as HCl, with the inorganic acid acting as a salting-out for [P_44414_]Cl resulting in a biphasic system at high acid concentrations [38,46]. The divergence in the underlying mechanism of phase separation in the [P_44414_]Cl + CH_3_COOH + H_2_O system is evidenced by its unusual closed “Type 0” phase diagram. “Type 0” diagrams are characterized by an immiscibility gap in the ternary region while all the binary mixtures are fully miscible [47]. The formation of aqueous biphasic systems using ionizable species, such as [P_44414_]Cl and acetic acids, leads to a complex system with potentially five species (four ionic + water). Specifically, the extent of carboxylate anion concentration depends on the acid pKa and solution pH. The pH of the studied systems never exceeded 3.5, implying that acetic acid (pKa = 4.75) can be primarily considered as the protonated non-ionic specie.

The appearance of a “type 0” biphasic system is unusual as such behavior is traditionally associated with polymer-IL type aqueous biphasic systems (ABS) [47,48,49]. The phase diagrams here reported differing drastically from the more traditional salt–salt mixtures as exemplified by the comparison of the [P_44414_]Cl + CH_3_COOH + H_2_O with the partial phase diagram of the corresponding ammonium acetate salt system in Figure 1B. The latter does not present a closed biphasic region unlike in the [P_44414_]Cl + CH_3_COOH + H_2_O system but rather a large immiscibility range-restricted at high salt concentrations by the solubility limit of NH_4_CH_3_COO. Comparison of the phase diagrams in Figure 1B contrasts the distinct salting-out influence exerted by the negatively charged acetate anion and the neutral protonated acetic acid. The inherent LCST properties of [P_44414_]Cl are maintained in the [P_44414_]Cl + CH_3_COOH + H_2_O system, Figure 1A, with an increase in the experimentally determined biphasic area with a temperature increase from 298 to 323 K. This contrasts with the upper critical solution temperature (UCST) behavior observed in polymer-IL type ABS where the immiscibility region decreases with increased temperature [47]. The “type 0” diagrams in Figure 1 represent an interesting separation approach for an integrated leaching/separation, where one can move from the pure mixture under the monophasic condition to a biphasic regime for separation through the simple addition of water and temperature manipulation.

For selected mixture points in the biphasic regime at 323 K, shown in Figure 1A, the concentration of the [P_44414_]^+^ cation and acid concentration were measured in both phases and reported in Table 1. Results indicate the preferential partition of the [P_44414_]Cl to one phase, with an almost quantitative distribution independent of the mixture point. Interestingly, acetic acid appears to distribute between the two phases, Table 1, despite its relative hydrophilicity with an octanol/water partition coefficients (K_OW_) of log(K_OW_) = −0.17 [50]. Such partitioning is noticeably different from that observed in salt-IL ABS where the IL and inorganic salt accumulate in opposite phases. The protonated carboxylic acid does not present a traditional salting-out inducing nature, yet the mixture of [P_44414_]Cl with CH_3_COOH presents a synergistic enhancement in hydrophobicity despite both individual components being fully miscible in water. Rather, the observed trend in Table 1 is reminiscent of the observed phase separation in [P_4444_]Cl-based hydrotrope system with gallic acid or vanillin as solute [51]. Such systems, above a certain solute concentration, phase-separated into a phase concentrated in both IL and solute and a diluted phase. Importantly for the reusability of the [P_44414_]Cl + CH_3_COOH systems, results in Table 1 indicate the loss of the original mixture composition. This can be partially mitigated in the presence of salts, promoting the partition of acetic acid to the IL-rich phase as shown in Table 1.

### 2.2. Mechanism of Phase Separation in the [P_44414_]Cl + CH_3_COOH + H_2_O System

To better understand the underlying mechanism of phase separation in the studied systems, the [P_44414_]Cl + CH_3_COOH system for a fixed molar ratio was simulated by classical all-atom (AA) molecular dynamics (MD) for various hydration levels from pure to diluted. The compositions of the systems simulated are summarised in Table 2 and CH_3_COOH was considered as fully protonated throughout.

**Figure 2:**
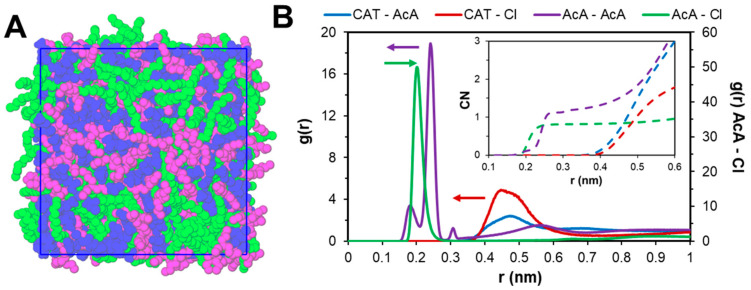
(**A**) Snapshot of the equilibrated [P_44414_]Cl + CH_3_COOH system with chloride anion and acetic acid in blue and C_4_ and C_14_ alkyl chains of [P_44414_]^+^ in purple and green respectively. (**B**) Radial distribution functions (RDFs) of the principal intermolecular interactions. The reference atom for [P_44414_]^+^ (CAT) was P whilst OH was taken for the carboxylic acid (AcA).

The structure of the [P_44414_]Cl + CH_3_COOH system in the absence of water, shown in Figure 2A, is visually characterized by a distinct nano-segregation into polar and apolar domains consisting of bi-continuous [P_44414_]^+^ aggregates and a percolating polar hydrogen-bonded network of chloride anion and acetic acid. Radial distribution function (RDF) analysis in Figure 2B indicates hydrogen-bonded CH_3_COOH···Cl^−^ and CH_3_COOH···OHOCCH_3_ carboxylic dimer as the predominant short-range interactions (≤0.3 nm), stabilized by longer-range IL cation-anion electrostatic interactions. The shielded nature of the [P_44414_]^+^ phosphorus charge center liberates the chloride anion to closely interact with the carboxylic acid and limits short-range Coulombic contributions. Furthermore, significant dispersive interaction between acetic acid and the butyl chains of the [P_44414_]^+^ polar head are observed, reaching inside the [P_44414_]^+^ aggregates as shown in the spatial distribution function representation in Figure S1.

**Figure 3:**
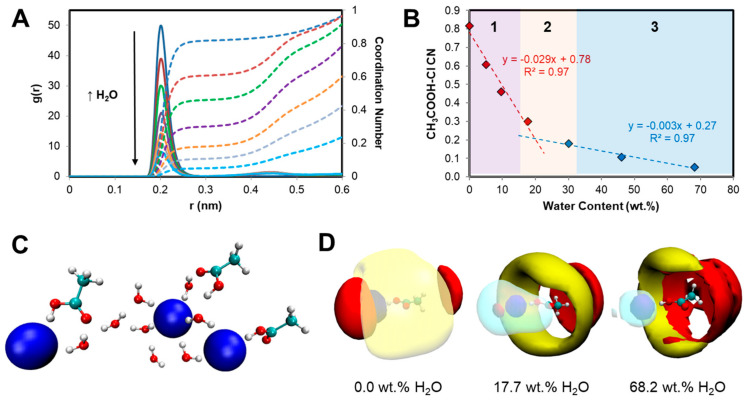
(**A**) RDFs of the CH_3_COOH···Cl^−^ with increasing water content (reference atoms for acetic acid were OH) and (**B**) corresponding coordination numbers (region 1 and 3 are monophasic whilst region 2 corresponds to the experimentally determined biphasic regime). (**C**) A representative snapshot of water-in-IL hydrated clusters for 5.1 wt.% H_2_O. (**D**) Spatial density function plots of the various system components around acetic acid for three water concentrations (Cl^−^—blue; [P_44414_]^+^—yellow; CH_3_COOH—red; H_2_O—cyan).

Upon addition of water, all intermolecular interactions identified in the pure mixture are systematically weakened, consistent with reported experimental and modeling of the influence of water on hydrophilic DES [33,52,53]. The systematic weakening of intermolecular interactions as each component becomes solvated is non-linear as exemplified in the CH_3_COOH···Cl^−^ RDF and resulting coordination number (CN) with increasing water content in Figure 3A,B. The defining interactions in the anhydrous mixture rapidly decrease with an increase in the water concentration and practically disappear as the system becomes sufficiently diluted. A clear break in the CH_3_COOHˑˑˑCl^−^ CN is observed at approximately 18.0 wt.% H_2_O, Figure 3B, with such behavior attributed to the change in system nanostructure from “water-in-IL” to “IL-in-water”. Under “water-in-IL” conditions for the [P_44414_]Cl + CH_3_COOH system ([H_2_O] ≤ 15.0 wt.%), gradual hydration of the system polar component is observed in which water molecules replace acetic acid and [P_44414_]^+^ around chloride anions forming hydrogen-bonded hydrated clusters as shown in Figure 3C. This shift in the molecular-scale segregation is also reflected in the CH_3_COOH···Cl^−^ domain analysis in Figure S2, calculated based on the Voronoi tessellation method [54,55], which indicates a systematic non-linear increase in the subset dispersivity upon water addition presenting a similar trend discontinuity as in Figure 3B. Relative to chloride anions, a significant fraction of the dispersive interaction between CH_3_COOH and [P_44414_]^+^ is maintained even at high water content, Figure S3. This is further evidenced in the 3D spatial density function (SDF) plots in Figure 3D projecting the most likely configurations of the various system components around acetic acid for three water concentrations. Whilst acetic acid–chloride (blue surface) hydrogen bonding is weakened due to intercalation of water (overlapping cyan surface), the presence of [P_44414_]^+^ (yellow surface) around acetic acid is maintained even under dilute conditions.

Interestingly, the observed discontinuity in Figure 3B or Figure S2 at approximately 18.0 wt.% coincides with the start of the experimentally determined biphasic regime. Such a trend points to the unequal “washing-out” and resulting solvophobic segregation of the components upon dilution, resulting in the phase separation of the system into a more apolar [P_44414_]Cl bi-continuous phase stabilized by adsorbed organic acid molecules and a water-rich phase. Further addition of water results in the complete solubilization of all components and the return of the monophasic system containing a [P_44414_]Cl micellar phase (≤40.0 wt.% IL) [38]. The presence of adsorbed neutral acetic acid onto the [P_44414_]Cl aggregate through acid alkyl moiety, shown in Figure S1, increases the dehydration of the aggregate surface and favors aggregate coalescence. Dynamic light scattering (DLS) analysis of [P_44414_]^+^ micelles (20.0 wt.% IL) in the presence of acetic acid, presented in Figure S4, shows the minimal micelle swelling with increasing acid concentration. This suggests that coacervation occurs rather through the nearing of neighboring micelles into a bi-continuous regime rather than through micelle-micelle fusion.

### 2.3. Metal Oxide Leaching in the [P_44414_]Cl + CH_3_COOH System

**Figure 4:**
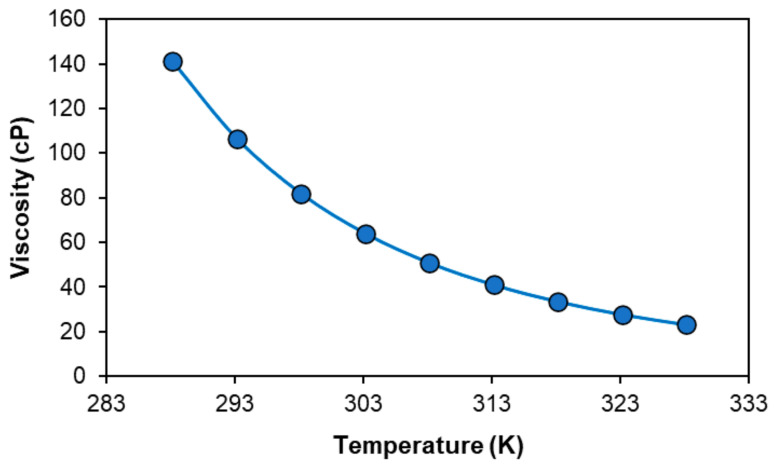
Viscosity of the 73.0 wt.% [P_44414_]Cl and 27.0 wt.% CH_3_COOH mixture as a function of temperature.

Having characterized its phase diagram and addressed the underlying mechanism of phase separation, the solubility of metal oxide in the [P_44414_]Cl + CH_3_COOH system is investigated. The solubility of three transition metal oxides CoO, NiO, Fe_2_O_3_ and one rare earth oxide Nd_2_O_3_ commonly found in nickel-metal hydride batteries (NiMH) in a 73.0 wt.% [P_44414_]Cl and 27.0 wt.% CH_3_COOH mixture at 323 K for 24 h is presented in Table 3. The mixture composition was selected such that a biphasic system could be obtained through the addition of water after metal oxide dissolution, with a 2.7 molar excess of acetic acid relative to the IL. At this composition, the mixture presents a moderate viscosity of 106.7 cP at 293 K which decreases to a manageable 27.7 cP at 323 K (Figure 4), an important consideration for the kinetics of mass transport during leaching. The water content of the “dry” mixture is of 1.3 wt.% H_2_O as measured by Karl–Fisher.

Total metal oxide solubility in the [P_44414_]Cl + CH_3_COOH system proceeds as CoO >> Nd_2_O_3_ > NiO > Fe_2_O_3_. The observed transition metal oxide solubility trend in the [P_44414_]Cl + CH_3_COOH mixture is consistent with that reported for choline chloride ([Ch]Cl) + CH_3_COOH DES as shown in Table 3. Interestingly, CoO presents a higher solubility in the [P_44414_]Cl + CH_3_COOH system compared to that in the corresponding 1:2 [Ch]Cl + CH_3_COOH solvent despite the lower chloride concentration of 1.68 mol kg^−1^ compared to 3.82 mol kg^−1^ respectively [42]. This is attributed to the greater hydrogen-bonded network, and therefore greater viscosity in choline chloride-based mixtures compared to the more aliphatic character of the [P_44414_]Cl + CH_3_COOH system. The presence of water in the mixture negatively influences the total CoO solubility. For a constant [P_44414_]Cl to CH_3_COOH molar ratio, the solubility of CoO slightly decreased from 0.349 mol L^−1^ in the “dry” mixture (containing 1.3 wt.% H_2_O) to 0.258 mol L^−1^ for a diluted mixture containing 30.0 wt.% H_2_O. This is most likely due to the increased solvation of the chloride anion limiting its chemisorption onto the active sites of the metal salt [42]. Despite the lower solubility of the transition metal oxides in the [P_44414_]Cl + CH_3_COOH system compared to mixtures incorporating stronger acids such as oxalic, lactic, maleic or toluene sulfonic acid,[13,42,57] the studied system presents a moderate selectivity for CoO. Nevertheless, additional separation is required to obtain a final cobalt product of suitable purity.

**Figure 5:**
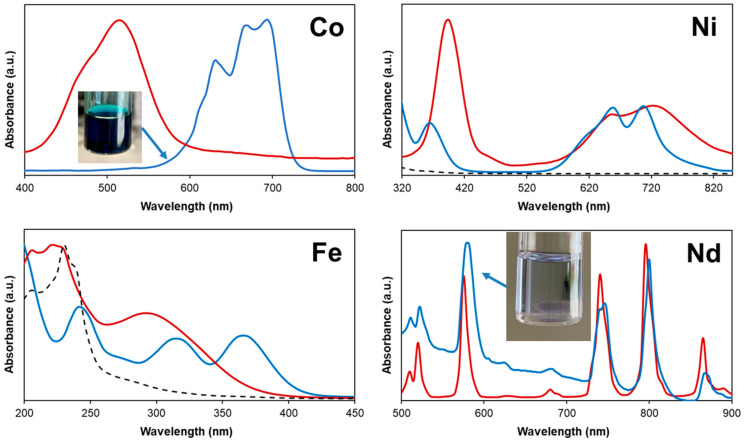
UV–vis spectra of the [P_44414_]Cl + CH_3_COOH system (73.0 wt.% [P_44414_]Cl and 27.0 wt.% CH_3_COOH) after metal oxide dissolution at 323 K for 24 h (blue line) and the corresponding spectra of the metal chloride salt in aqueous solution (red line). The dashed line corresponds to the spectra of the [P_44414_]Cl + CH_3_COOH mixture in the absence of metals.

Metal oxide solubility in mixtures of halogenated salts with Brønsted acids was shown to be dependent on its Gibbs energy of formation (∆*_f_G^o^*), explaining the greater solubility of CoO compared to Fe_2_O_3_ (Table 3) [42]. However, such a justification does not explain the low solubility of NiO or the unexpectedly high solubility of Nd_2_O_3_, the most stable of the tested oxides. Although the ∆*_f_G^o^* values of NiO and CoO are similar, the significantly greater solubility of CoO in the [P_44414_]Cl + CH_3_COOH mixture is assigned to the more chlorophilic nature of Co(II) compared to Ni(II). This is evidenced when comparing the UV–vis spectra of the metal oxide saturated [P_44414_]Cl + CH_3_COOH solution to the corresponding spectra of the salt in water, Figure 5. The CoO saturated [P_44414_]Cl + CH_3_COOH solution displayed a deep blue color characteristic of the tetrahedral CoCl_4_^2-^ complex. Similarly, the UV–vis spectrum of Fe(III) in aqueous solution differs from that in the IL mixture after leaching, with the latter indicating the existence of Fe(III) as the FeCl_4_^−^ specie [58]. Whilst most transition metals form tetrachloride species in chloride-containing ionic solvents and mixtures, the speciation of nickel was found to differ [5]. Qualitative comparison of the spectrum of Ni(II) in water and in the [P_44414_]Cl + CH_3_COOH mixture after leaching confirms the stability of the Ni(II) octahedral geometry. The absorption bands of aqueous [Ni(H_2_O)_6_]^2+^ at 395 nm and 720 nm are blue-shifted to 365 nm and 705 nm respectively, whilst the relative absorption of the band at 365 nm (^3^A_2g_→^3^T_1g_(P) transition) decreases in the mixture and a new shoulder appears at 615 nm. The UV-vis spectrum of Ni(II) in the mixture are inconsistent with that of Ni(II) in concentrated chloride solutions in which a systematic red-shift of the bands is observed as chloride progressively replaces H_2_O within the first coordination shell [59]. These differences confirm the more complex Ni(II) coordination in the [P_44414_]Cl + CH_3_COOH mixture and its probable interaction with neutral acetic acid. ILs incorporating carboxylic acid moieties as well as mixtures containing carboxylic acids, such as ethylene glycol + maleic acid, were shown to efficiently solubilize rare earth oxides despite the latter’s stability (∆*_f_G^o^*(Nd_2_O_3_) = −1854.2 kJ mol^−1^) [57,60,61]. In such solutions, a small amount of water was proven to enhance the oxide solubility by facilitating the acid deprotonation [60,61]. The water content of 1.3 wt.% H_2_O in the [P_44414_]Cl + CH_3_COOH mixture could partially explain the observed Nd_2_O_3_ solubility. The UV–vis spectrum for Nd(III) in Figure 5 is characterized by a number of Laporte-forbidden f–f transitions, with the spectrum of Nd(III) in the mixture red-shifted relative to its aqueous spectrum suggesting that Nd(III) is complexed with acetate anions. This appears as the likeliest scenario considering the far greater complexation constant of neodymium with acetate compared to chloride ligands [62,63].

### 2.4. Application of the [P_44414_]Cl + CH_3_COOH System to Waste NiMH Battery

**Figure 6:**
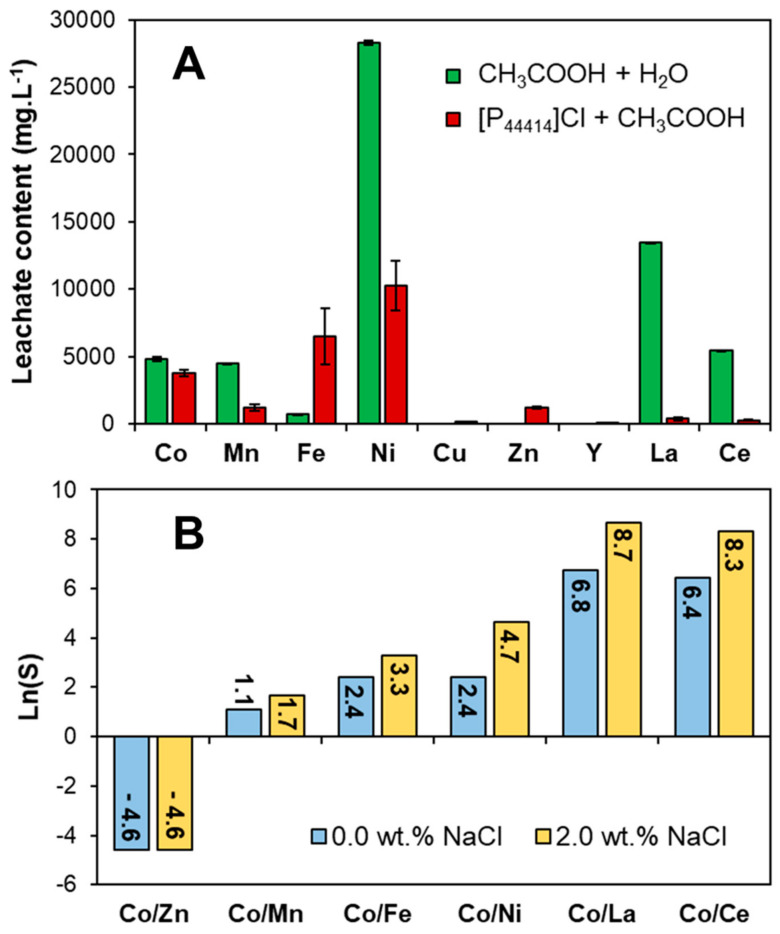
(**A**) Metal concentration in the NiMH battery leachate composed of 27.0 wt.% CH3COOH aqueous solution and in the 73.0 wt.% [P_44414_]Cl + 27.0 wt.% CH_3_COOH mixture (t = 24 h, *T* = 323 K, S/L = 0.1). (**B**) Co(II) selectivity (S) in the biphasic [P_44414_]Cl + CH_3_COOH + H_2_O from the principal metals in the NiMH leachate. The total composition of systems **1** and 1* are provided in Table 1; individual metal distribution coefficients are available in Table S1.

Based on the results presented above the [P_44414_]Cl + CH_3_COOH system is applied in the integrated process of leaching and separation of Co(II) from real waste NiMH battery black mass obtained from an industrial recycler (Recupyl, Grenoble, France). NiMH battery black mass is characterized by a high Ni(II) concentration, as well as lesser quantities of rare earth elements, Co(II), Mn(II) and Zn(II). Impurities such as Fe(III) are often also encountered [64]. To minimize acid–base neutralization from residual KOH battery electrolyte, the black mass was rinsed with deionized water until a stable pH was obtained prior to leaching studies. Two systems were evaluated for the leaching of Co(II) from the complex waste matrix: (i) an aqueous solution of 27.0 wt.% CH_3_COOH, (ii) 73.0 wt.% [P_44414_]Cl + 27.0 wt.% CH_3_COOH. Co(II) leaching in the [P_44414_]Cl + CH_3_COOH mixture is comparable to that using an aqueous acetic acid solution but present a greater selectivity, with a lower Ni(II), Mn(II) and rare earth element concentration, as shown in Figure 6A. However, an increase in Fe(III) and Zn(II) leaching is observed due to the higher chloride concentration resulting from the lower water content.

Following the leaching of the black mass for 24 h at 323 K, the saturated [P_44414_]Cl + CH_3_COOH mixture was diluted either with water or a 2.0 wt.% NaCl until a biphasic system was obtained with composition **1** and 1* in Table 1 respectively. The distribution ratio (D) for a given metal M^n+^ between both system phases is defined in Equation (1), whilst the system selectivity (S) for Co(II) is defined according to Equation (2):

(1) $$D_{M}=\frac{{\left[M^{n+}\right]}_{\mathrm{IL}}}{{\left[M^{n+}\right]}_{H_{2}O}}$$

(2) $$S_{\frac{\mathrm{Co}}{M}}=\frac{D_{\mathrm{Co}}}{D_{M}},$$

where the subscript IL and H_2_O indicate the respective IL-rich and water-rich phases and [M^n+^] is the concentration of metal M^n+^. The distribution coefficients and extraction percentages obtained in system **1** and 1* are presented in Table S1 whilst the Co(II) selectivity is shown in Figure 6B. The addition of 2.0 wt.% NaCl improves both the Co(II) partition to the IL-rich phase, increasing D_Co_ from 0.10 to 0.49, as well as the system selectivity (Figure 6B). Although the [P_44414_]Cl + CH_3_COOH system presents a limited D_Co_, this can be further improved through the addition of chloride salts to promote the formation of the anionic chlorocobalt complex. Optimal Co(II) extraction by phosphonium-based ILs occurs at around 6.0 to 8.0 mol L^−1^ of chloride, far above that in the studied system [65,66,67]. Addition of chloride salts presents the added benefit of (i) increasing the partition of CH_3_COOH to the IL-rich phase (*cf.* Table 1) and (ii) extending the system biphasic region [65].

According to the results in Figure 6B as well as the UV–vis spectra in Figure 5, metal partition in the [P_44414_]Cl + CH_3_COOH biphasic system relies on the formation of neutral or anionic chlorometallate complexes and their subsequent extraction to the IL-rich phase via an ion-pairing or anion-exchange mechanism. Metal cations known not to form the required anionic complexes with chloride ligands such as Ni(II) and the lanthanides La(III) and Ce(III) are not extracted, resulting in a high Co(II) selectivity relative to these metals, Figure 6B. Conversely, the system presents moderate to no selectivity for Co(II) against the metals Zn(II), Cu(II) and Mn(II) as these form anionic complexes at lower (Zn(II) and Cu(II)) or similar (Mn(II)) chloride concentrations as Co(II) [65,66,67]. Overall, a similar Co(II) selectivity trend is observed in the [P_44414_]Cl + CH_3_COOH system compared to solvent-extraction using quaternary ammonium or phosphonium extractant with the notable extraction of Fe(III) [65,66,67]. In contrast to the preferential extraction of Fe(III) over Co(II) by the hydrophobic [P_66614_]Cl from chloride media, the [P_44414_]Cl + CH_3_COOH presents an interesting selectivity towards Co(II) with S_Co/Fe_ = 27.1 most likely due to the competing complexation of acetic acid with chloride ligands. The [P_44414_]Cl + CH_3_COOH system can selectively recover Co(II) from Ni(II), Fe(III) and the lanthanides from a complex waste matrix. Co(II) was enriched from 15.5 wt.% of the total metal content in the initial leachate to 54.7% in the IL-rich phase after phase separation through water addition. Zn(II) was the major impurity in the IL-rich phase, accounting for 23.6% of the extracted metal content.

Although some issues still need to be overcome to increase the applicability of the proposed system, namely the low Co(II) distribution and the cost and toxicity of [P_44414_]Cl IL, this work provides guidelines for the development of responsive non-aqueous mixtures for the integrated leaching and separation of critical metals from complex waste streams. The close to fourfold concentration of Co(II) in the mixture through the simple addition of water greatly simplifies downstream purification, opening the possibility for Co(II) recovery by electrodeposition [65]. This proof of concept demonstrates how the judicious mixture of an IL with an organic acid can result in the selective “one-pot” leaching and solvent extraction separation of target metals from multiple impurities present in a real waste matrix through the simple addition of water as a counter-solvent. This contrasts with leaching processes using aqueous acidic solutions or hydrophilic eutectic solvents from which additional separation stages requiring further solvent consumption are required.

## 3. Methodology

### 3.1. Reagent and Instrumentation

The ionic liquids [P_4444_]Cl (95.0 wt.%), [P_44414_]Cl (95.0 wt.%) were obtained from Cytec industries, [N_4444_]Cl (97.0 wt.%) from Sigma-Aldrich (St. Louis, MO, USA)and [C_14_mim]Cl (98.0 wt.%) from Iolitec (Heilbronn, Germany). Citric acid (99.5 wt.%) was obtained from Panreac, lactic acid (92.0 wt.%) from Riedel de Haen and acetic acid (99.9 wt.%) from Fisher Scientific (Waltham, MA, USA). Neodymium (III) oxide (99.99%), cobalt (II) oxide (95%) and iron (III) oxide (98%) were purchased from Alfa Aesar (Haverhill, MA, USA) whilst nickel (II) oxide (99.99%) and the gallium standard (1000 mg L^−1^) were acquired from Sigma-Aldrich. Ammonium acetate (98.0 wt.%) and sodium chloride (99.5 wt.%) were obtained from Merck (Darmstadt, Germany) and and Fisher Scientific, respectively. All chemicals were used as received. Ultrapure deionized water was used in all experiments and obtained from an ultra-filtration system by reverse osmosis and subsequently passed through a Milli-Q plus 185 water purification apparatus (18.2 MΩ cm to 298 K). All systems were gravimetrically prepared through careful weighing of each component using an analytical scale (Precisa gravimetries AG, uncertainty 0.0001 g). The cation of the ionic liquid ([P_44414_]^+^) was quantified by ^1^H-NMR (Bruker Avance III, 400 Hz (Billerica, MA, USA)) using as internal standard a benzene probe of known concentration. Acid-base titration was used to determine acid contraction in the phases (Titroline 6000 pH meter; 0.1%). Karl Fischer titration was used to determine the amount of water (Titroline 7500 KF Trace; 0.15%). A thermostatic bath (ME-18 V Visco-Thermostat, Julabo, (Seelbach, Germany) ±0.1 K) was used for temperature control. Viscosity was determined using a viscometer-densimeter SVM 3000 Anton Paar Rotary Stabinger (Graz, Austria), with a temperature uncertainty of ±0.1 K and a relative uncertainty of dynamic viscosity of ±0.35%. Aggregate size measurements were made by dynamic light scattering (DLS) (Malvern Zetasizer Nano-ZS) measurements. For measurements of metals has been used Picofox S2 (Bruker Nano (Billerica, MA, USA)) total reflection X-ray fluorescence spectrometer with a molybdenum X-ray source. The voltage of the X-ray tube was 50 kV and the current 600 μA. All carriers were first pretreated with 10 μL of silicon in isopropanol solution and dried at 353 K for 30 min. Ten microliters of each solution containing the metals and standard of Ga were added onto a clean carrier and dried on a hot plate at 60 °C for 15 min.

### 3.2. Determination and Characterisation of [P_44414_]Cl + Organic Acid + H_2_O Systems

In a first instance, a screening of four different ILs, namely [N_4444_]Cl, [P_4444_]Cl, [P_44414_]Cl and [C_14_mim]Cl, with various water-soluble organic acids in aqueous solutions was performed at 323 K to identify potential biphasic mixtures. For each IL + organic acid + H_2_O system, two initial binary mixture compositions were selected to which the third component was titrated until the system became turbid. Initial mixture compositions were of 75.0 wt.% IL in water and a 50:50 wt.% IL + organic acid (including the initial acid water content), respectively.

Based on the screening results, the phase diagram of the [P_44414_]Cl + CH_3_COOH + H_2_O system was experimentally determined using a combination of the cloud point titration method and turbidimetric method performed in a temperature-controlled thermostatic bath. The cloud point, representing the solution turbidity caused by phase demixing, was visually detected when a mass of acid or aqueous salt solution was added dropwise over an aqueous IL solution of known mass, and a known mass of water was added until the solution became clear again, this process being repeated several times. The [P_44414_]Cl and CH_3_COOH concentration ranges probed in this study are 2.0 to 75.0 wt.% IL and 0.0 to 30.0 wt.% CH_3_COOH respectively. Additionally, to delimit the “type 0” phase diagram of [P_44414_]Cl + CH_3_COOH + H_2_O, mixing points of known composition concentrated in organic acid and IL were prepared and a known mass of water was added dropwise in each tube until the appearance of cloudiness. All mixing points were made in triplicate.

The composition of the equilibrium phases for selected mixture points at 323 K was experimentally determined. The mixtures were gravimetrically prepared, agitated and left to separate for 12 h under temperature control. The equilibrium phases were carefully separated to determine the respective concentration of IL, CH_3_COOH and water in each phase. All mixing points were made in duplicate. DLS measurements were performed to evaluate the variation in [P_44414_]^+^ aggregate size as a function of CH_3_COOH concentration. A constant [P_44414_]Cl concentration of 20.0 wt.% was used in all DLS measurements, corresponding to the micellar regime of this surfactant [38]. Samples were irradiated with a HeNe laser (λ = 565 nm) and the intensity fluctuations of the scattered light were detected at a backscattering angle of 173° to generate an autocorrelation function. The autocorrelation function was corrected from the default settings for deionized water by adjusting for the solution viscosity (3.586 cP) and refractive index (1.3324). The cumulant analysis of this function provided by the software DTS v 7.03 yielded the particle size and the distribution width. Each composition tested was analyzed three times to ensure the formation of stable aggregates. Ultrapure water was used in all analyses.

### 3.3. Dissolution of Metal Oxides and Separation

The solubilities of different transition metal and rare earth oxides (NiO, CoO, Fe_2_O_3_, Nd_2_O_3_) in the [P_44414_]Cl + CH_3_COOH (73.0 wt.% IL, 27.0 wt.% CH_3_COOH) system were experimentally determined through the addition of an excess of metal oxide to 2.0 g the mixture for 24 h at 323 K and 500 rpm. The leachate was subsequently isolated by centrifugation and appropriately diluted using deionized water prior to metal concentration analysis by UV–vis and TXRF.

The system [P_44414_]Cl + CH_3_COOH (73.0 wt.% IL, 27.0 wt.% CH_3_COOH) was subsequently used to leach and separate the metals contained in the real waste NiMH battery black mass. NiMH battery black mass was kindly provided by an industrial recycler (Recupyl, Grenoble, France) and washed with deionized water prior to use to remove residual KOH electrolyte. To 1.8 g of the IL + acid mixture was added 0.18 g of waste NiMH black mass and left under stirring at 500 rpm for 24 h at 323 K. Following leaching, the saturated [P_44414_]Cl + CH_3_COOH mixture was diluted with 0.5 g of H_2_O or a 2.0 wt.% aqueous NaCl solution until a biphasic system was obtained. The systems were centrifugated to speed up the phase separation. The phases were subsequently diluted using deionized water prior to metal concentration analysis by UV–vis and TXRF. The same leaching conditions, namely 500 rpm for 24 h at 323 K and a solid-to-liquid ratio of 0.1, were employed to evaluate the leaching of metals from 0.18 g of the black mass using an aqueous solution of 27.0 wt.% CH_3_COOH.

### 3.4. Molecular Dynamic Simulations of the [P_44414_]Cl + CH_3_COOH+ H_2_O System

All simulations were carried out with the Gromacs 5.1 package [68] within the NpT ensemble by adopting the leapfrog algorithm to integrate the equations of motion with a time step of 2 fs at a fixed temperature (323 K) and pressure (1 bar) [69]. The energy contributions in the potential energy function for bonded interactions were bond stretching, angle bending and dihedral torsion, whilst non-bonded inter-actions were modelled by Lennard–Jones (LJ) and Coulombic terms. The force-switch van der Waals potential modifier was employed for LJ, where the energy decays smoothly to zero between 0.9 and 1.2 nm, while long-range Coulombic interactions were evaluated by particlemesh Ewald (PME) [70], up to a cut-off radius of 1.2 nm. The temperature and pressure were fixed at 323 K and 1 bar through the Nose–Hoover thermostat [71] and the Parrinello–Rahman barostat [72], respectively. All bonds were constrained by the LINCS algorithm during the simulations [73]. The force field parameters for [P_44414_]Cl were taken from the IL OPLS-AA all-atom force field developed by Canongia Lopes and Padua [74] and water molecules were represented by the SPC/E model [75]. In all simulations, acetic acid was considered as protonated and its parameter was taken from the OPLS-AA forcefield. Cubic boxes with periodic boundary conditions were used placing all the molecules randomly, followed by an equilibrium process. All systems contain 200 [P_44414_]Cl ion pairs, 580 acetic acid molecules and a varied number of water molecules. The composition of the system simulated are summarised in Table 2. For all simulations, the following equilibration protocol was followed: an energy minimization step using the steepest descent algorithm to prevent short-range contacts between atoms prior to two short equilibrium runs in the NVT and NpT ensembles, respectively. Afterwards, all systems were run for 100 ns of simulation time in the NpT ensemble. Simulation outputs were visualized using the VMD software package [76] and the spatial distribution functions (SDF) and domain analysis were analyzed using the TRAVIS package [54,55].

## 4. Conclusions

The phase diagram of the [P_44414_]Cl + CH_3_COOH + H_2_O system is studied here for the first time, it being shown that the system presents an unusual “type 0” closed phase diagram and an LCST type behavior. MD simulations of the [P_44414_]Cl + CH_3_COOH system indicate that the formation of the biphasic regime is due to a “washing-out” phenomenon upon addition of water, driven by water nanostructuring ultimately resulting in solvophobic segregation of the [P_44414_]Cl with acetic acid. This behavior is markedly different from conventional IL-salt, polymer-IL or even IL-inorganic acid aqueous biphasic system [65]. The [P_44414_]Cl + CH_3_COOH system, in the absence of additional water, presents a good solubility for CoO as well as for Nd_2_O_3_. UV–vis analyses indicate varied metal speciation in the mixture from anionic chlorometallate complexes for Co(II) and Fe(III), acetate Nd(III) specie to more complex speciation in the case of Ni(II).

The [P_44414_]Cl + CH_3_COOH system was applied to the treatment of real waste NiMH battery black mass, with the integration of the leaching and separation steps achieved by the simple addition of water to the leachate yielding a biphasic regime that allows one of the phases to become enriched in Co(II). Both the leaching and metal separation steps are selective for Co(II) against Ni(II), Mn(II) and the rare earth elements. Furthermore, extraction in the [P_44414_]Cl + CH_3_COOH presents an interesting selectivity for Co(II) against Fe(III). However, additional separation steps are required for the separation of Co(II) from Zn(II) and Mn(II). The close to fourfold concentration of Co(II) from leaching to separation in a single solution through the simple addition of water greatly simplifies downstream purification and provides a guideline for metal process intensification in other alternative solvents such as DES.

## Figures and Tables

**Table 1:** Quantification of phase composition for the aqueous biphasic system composed of [P_44414_]Cl and CH_3_COOH at 313 K (TLL—Tie Line Length). The mixture point 1* has the same initial [P_44414_]Cl and CH_3_COOH concentration as mixture point 1 plus 2.00 wt.% NaCl.

Mixture Point	Total Composition (wt.%)			Top Phase (wt.%)				Bottom Phase (wt.%)				TLL
	IL	Acid	H_2_O	IL	Acid	H_2_O	Vol (mL)	IL	Acid	H_2_O	Vol (mL)	
1	55.00	20.00	25.00	49.02	12.00	38.98	2.0	1.41	25.31	73.27	0.4	49.43
1*	55.00	20.00	23.00	64.81	26.15	9.04	1.9	0.01	13.72	86.27	0.5	-
2	50.00	25.00	25.00	69.77	24.08	6.15	2.0	0.10	30.72	69.18	0.4	69.99

**Table 2:** [P_44414_]Cl + CH_3_COOH + H_2_O system composition for all AA simulations at 323 K. All systems contain 200 [P_44414_]Cl ion pairs and CH_3_COOH molecules with an increasing number of water molecules (n(H_2_O)).

System	n(H_2_O)	[IL] (wt.%)	[CH_3_COOH] (wt.%)	[H_2_O] (wt.%)	Experimental Regime
(1)	0	71.4	28.6	0.0	Monophasic
(2)	363	67.8	27.1	5.1	Monophasic
(3)	725	64.5	25.8	9.7	Monophasic
(4)	1450	58.8	23.5	17.7	Biphasic
(5)	2900	50.0	20.0	30.0	Biphasic
(6)	5800	38.4	15.4	46.2	Monophasic
(7)	14,500	22.7	9.1	68.2	Monophasic

**Table 3:** Gibbs energy of formation (*∆_f_G^o^*) and solubility for selected metal oxides in [P_44414_]Cl+CH_3_COOH after 24 hr at 323 K compared to that in choline chloride ([Ch]Cl)) + CH_3_COOH (1:2) DES.

Metal Oxide	*∆_f_G^o^* (kJ·mol^−1^)	Solubility (mol·L^−1^)	Solubility (mol·L^−1^)
		[P_44414_]Cl + CH_3_COOH	[Ch]Cl + CH_3_COOH ^a^
CoO	−214.1 ^a^	0.349 ± 0.030	~0.10
NiO	−211.7 ^a^	0.028 ± 0.001	~0.01
Fe_2_O_3_	−742.8 ^a^	0.015 ± 0.001	<0.01
Nd_2_O_3_	−1854.2 ^b^	0.036 ± 0.002	-

^a^ [42], ^b^ [56].

## Supplementary Materials

Figure S1: 3D spatial density function (SDF) plot of the [P_44414_]Cl + CH_3_COOH +H_2_O system for two different water content; Figure S2: Domain analysis of the CH_3_COOH+Cl^−^ subset based on the Voronoi tessellation method; Figure S3: Standardised coordination numbers (CNs) between [P_44414_]^+^ and CH_3_COOH or Cl^−^ as a function of the water content; Figure S4: Average diameter of [P_44414_]^+^ aggregates (20.0 wt.% IL) in the presence of acetic acid as estimated by dynamic light scattering; Table S1: Distribution coefficients in the biphasic [P_44414_]Cl + CH_3_COOH + H_2_O of the principal metals in the NiMH leachate.
