# Supplementary material for: Integrated Leaching and Separation of Metals Using Mixtures of Organic Acids and Ionic Liquids
Source: Molecules. 2020 Nov 27;25(23):5570. doi: 10.3390/molecules25235570 (PMC7729566; doi:10.3390/molecules25235570)
Supplement: Supplementary file 1 [file molecules-25-05570-s001.pdf]

SUPPORTING INFORMATION

Integrated Leaching and Separation of Metals Using Mixtures of Organic Acids and Ionic Liquids

Silvia J. R. Vargas, Helena Passos, Nicolas Schaeffer * and João A. P. Coutinho

CICECO-Aveiro Institute of Materials, Department of Chemistry, University of Aveiro, 3810-193 Aveiro, Portugal; silvia.vargas@ua.pt (S.J.R.V.); hpassos@ua.pt (H.P.); jcoutinho@ua.pt (J.A.P.C.)

* Correspondence: nicolas.schaeffer@ua.pt

FIGURES

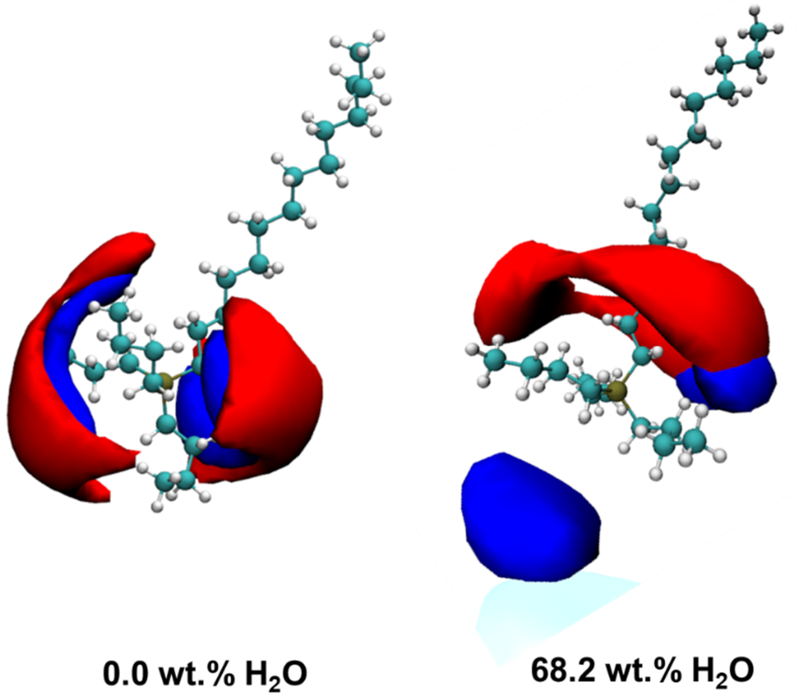

Figure 1. 3D spatial density function (SDF) plot of the $[P_{44414}]Cl$ + CH_3COOH +H_2O system for two different water content projecting the most probable configurations of acetic acid (red surface) and chloride anion (blue surface) around the reference $[P_{44414}]^+$ cation. System compositions are provided in **Table 2** of the manuscript.

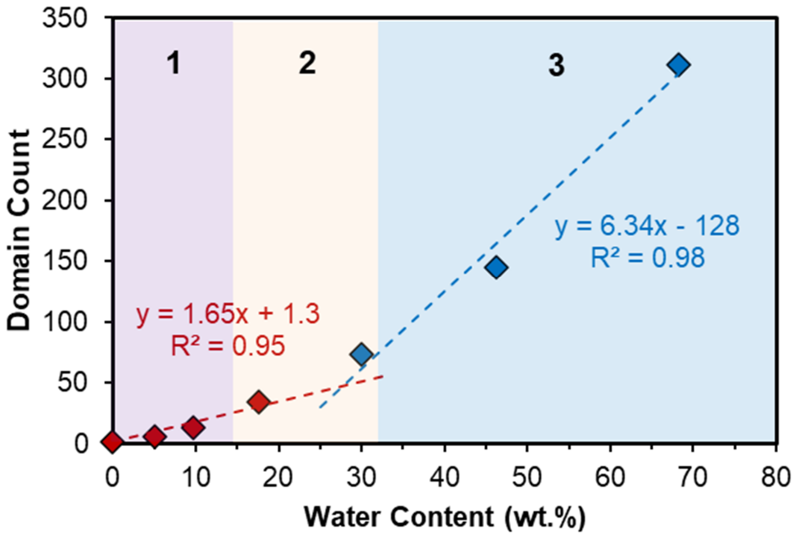

Figure 2. Domain analysis of the $CH_3COOH+Cl^-$ subset based on the Voronoi tessellation method (region 1 and 3 are monophasic whilst region 2 corresponds to the experimentally determined biphasic regime).[1,2] A value of 1 implies the molecules in the subset form a continuous aggregate, whilst a larger value is indicative of a dispersed subset. System compositions are provided in **Table 2** of the manuscript.

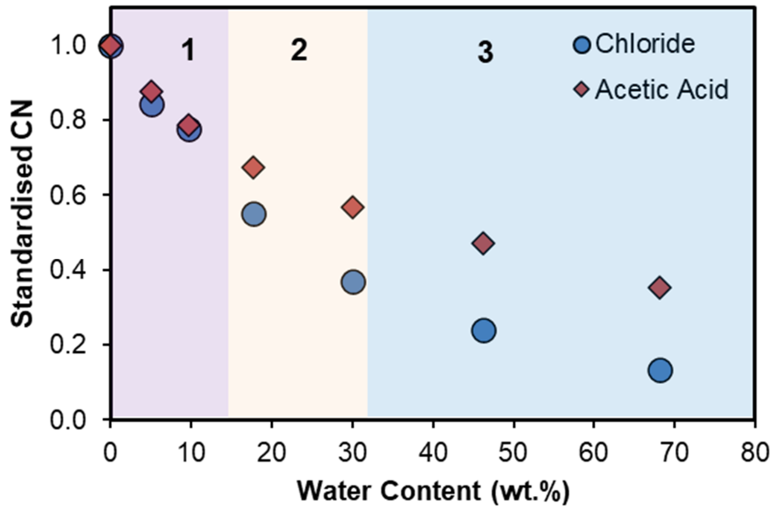

Figure 3. Standardised coordination numbers (CNs) between $[P_{44414}]^+$ and CH_3COOH or Cl^- as a function of the water content (region 1 and 3 are monophasic whilst region 2 corresponds to the experimentally determined biphasic regime). The central P atom and the OH atoms were selected as the reference atoms for $[P_{44414}]^+$ and CH_3COOH respectively. CNs were standardised relative to anhydrous $[P_{44414}]Cl$ + CH_3COOH system. System compositions are provided in **Table 2** of the manuscript.

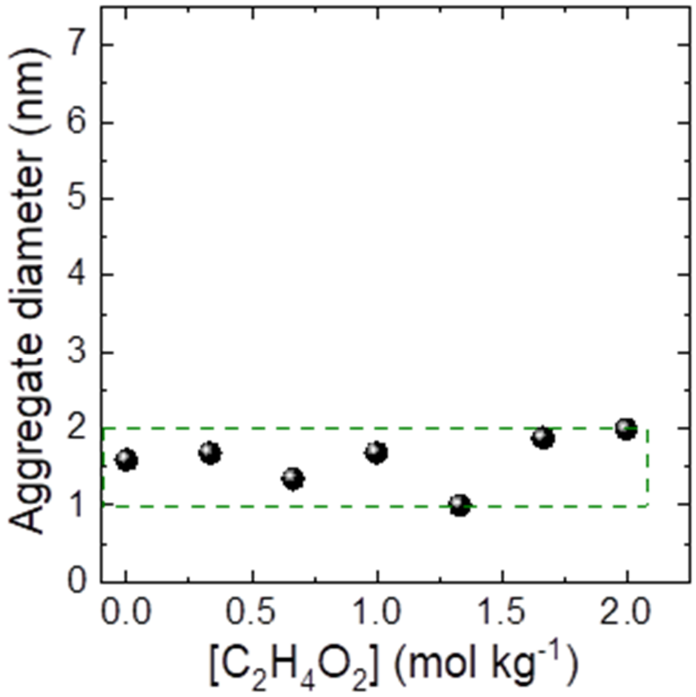

Figure 4. Average diameter of $[P_{44414}]^+$ aggregates (20.0 wt.% IL) in the presence of acetic acid as estimated by dynamic light scattering.

TABLES

Table 1. Distribution coefficients (D_M) and extraction percentages ($\%E_M$) in the biphasic $[P_{44414}]Cl + CH_3COOH + H_2O$ system of the principle metals in the NiMH leachate. The total composition of systems **1** and **1*** are provided in **Table 1** of the manuscript.

Metal	System 1 (0.0 wt.% NaCl)		System 1* (2.0 wt.% NaCl)	
	D_M	$\%E_M$	D_M	$\%E_M$
Co	0.10	32.21	0.49	70.93
Mn	0.03	13.95	0.09	31.74
Fe	0.01	4.16	0.02	8.26
Ni	0.01	4.05	>0.01	2.29
Zn	5474.89	99.99	4910.00	99.92
Y	> 0.01	0.27	> 0.01	0.28
La	> 0.01	0.06	> 0.01	0.04
Ce	> 0.01	0.08	> 0.01	0.06

REFERENCES

1. Brehm, M.; Kirchner, B. TRAVIS - A free analyzer and visualizer for monte carlo and molecular dynamics trajectories. *J. Chem. Inf. Model.* **2011**, *51*, 2007–2023, doi:10.1021/ci200217w.
2. Brehm, M.; Weber, H.; Thomas, M.; Hollóczki, O.; Kirchner, B. Domain Analysis in Nanostructured Liquids: A Post-Molecular Dynamics Study at the Example of Ionic Liquids. *ChemPhysChem* **2015**, *16*, 3271–3277, doi:10.1002/cphc.201500471.
